# Towards understanding residual and dilated dense neural networks via convolutional sparse coding

**DOI:** 10.1093/nsr/nwaa159

**Published:** 2020-07-13

**Authors:** Zhiyang Zhang, Shihua Zhang

**Affiliations:** National Center for Mathematics and Interdisciplinary Sciences, Center for Excellence in Mathematical Science, Key Laboratory of Random Complex Structures and Data Science, Academy of Mathematics and Systems Science, Chinese Academy of Sciences, Beijing 100190, China; School of Mathematical Sciences, University of Chinese Academy of Sciences, Beijing 100049, China; National Center for Mathematics and Interdisciplinary Sciences, Center for Excellence in Mathematical Science, Key Laboratory of Random Complex Structures and Data Science, Academy of Mathematics and Systems Science, Chinese Academy of Sciences, Beijing 100190, China; School of Mathematical Sciences, University of Chinese Academy of Sciences, Beijing 100049, China; Center for Excellence in Animal Evolution and Genetics, Chinese Academy of Sciences, Kunming 650223, China; Key Laboratory of Systems Biology, Hangzhou Institute for Advanced Study, University of Chinese Academy of Sciences, Chinese Academy of Sciences-, Hangzhou 310024, China

**Keywords:** convolutional neural network, convolutional sparse coding, residual neural network, mixed-scale dense neural network, dilated convolution, dense connection

## Abstract

Convolutional neural network (CNN) and its variants have led to many state-of-the-art results in various fields. However, a clear theoretical understanding of such networks is still lacking. Recently, a multilayer convolutional sparse coding (ML-CSC) model has been proposed and proved to equal such simply stacked networks (plain networks). Here, we consider the initialization, the dictionary design and the number of iterations to be factors in each layer that greatly affect the performance of the ML-CSC model. Inspired by these considerations, we propose two novel multilayer models: the residual convolutional sparse coding (Res-CSC) model and the mixed-scale dense convolutional sparse coding (MSD-CSC) model. They are closely related to the residual neural network (ResNet) and the mixed-scale (dilated) dense neural network (MSDNet), respectively. Mathematically, we derive the skip connection in the ResNet as a special case of a new forward propagation rule for the ML-CSC model. We also find a theoretical interpretation of dilated convolution and dense connection in the MSDNet by analyzing the MSD-CSC model, which gives a clear mathematical understanding of each. We implement the iterative soft thresholding algorithm and its fast version to solve the Res-CSC and MSD-CSC models. The unfolding operation can be employed for further improvement. Finally, extensive numerical experiments and comparison with competing methods demonstrate their effectiveness.

## Teaser

From the view of convolutional sparse coding, we build mathematically equivalent forms of two advanced deep learning models including residual and dilated dense neural networks with skip connections.

## INTRODUCTION

Nowadays, neural networks have become effective techniques in many fields, including computer vision, natural language processing, bioinformatics, etc. Their predecessor perceptron was proposed by Rosenblatt in 1958 [1]. However, the perceptron is too simple to solve the Exclusive OR (XOR) problem. To tackle more complex problems, multilayer perceptron (MLP) was proposed. Neural networks can be seen as generalized MLPs with a series of special operations. The activation functions (e.g. Sigmoid, Tanh and the rectified linear unit (ReLU) [2]) have been used for computing the outputs of hidden layers in neural networks to simulate the thresholding activation of neurons in the human brain.

Convolution is another important operation used for processing data that has a known, gridlike topology. For example, time-series data can be regarded as one-dimensional grid data and an image can be thought of as a two-dimensional grid of pixels. Convolution operation simulates human eyes, capturing features locally and scanning globally. The range within which features are captured is called the receptive field [3]. Early convolutional neural network (CNN) related architectures proposed in the 1980s [4–6] greatly inspired current deep CNNs (DCNNs).

Rapid improvements in hardware and public availability of highly optimized software [7–9] make it possible to train a neural network with a large number of parameters. AlexNet [10] is such a classical CNN architecture, which draws attention to DCNNs. Deeper networks have a stronger ability to fit complex distributions, so it is easier to achieve better performance than ever before. ‘The deeper, the better’ becomes a belief [11]. However, DCNNs are hard to train because of diverse optimization issues. The problem of vanishing or exploding gradients [12,13] is a notorious problem that has been addressed in two novel ways: normalized initialization [13,14] and batch normalization [15]. When deep neural networks are trained using these two techniques, the degradation phenomenon that deep networks achieve lower accuracy than shallow networks is exposed [16]. The residual neural network (ResNet) [16] is a special architecture with skip connections that tackles this phenomenon. Difficulties have been resolved, but the optimization issues behind the degradation phenomenon are still not clear.

The densely connected CNN [17] and mixed-scale dense convolutional neural network (MSDNet) [18] are also well-known architectures with skip connections. Usually, they have fewer parameters to overcome the overfitting issue. Besides, there are many other architectures and methods, such as Dropout [19], VGG [11], GoogLeNet [20], R-CNN [21], YOLO [22] and FCN [23]. All these have become very important solutions in neural networks.

We still however do not understand the principle of CNNs clearly. All the above successes are mainly based on empirical exploration. A clear and profound theoretical understanding of such neural networks is still lacking. On the one hand, architectures with excellent performance are hard to strictly interpret. On the other hand, the design of architectures mainly depends on intuition or inspiration. The lack of theory is currently a key problem that limits further development of neural networks. This situation brings uncertainty when people apply neural networks to some challenging fields, such as self-driving, medical diagnosis and identity recognition.

Recently, the connection between convolutional sparse coding (CSC) [24,25] and CNNs has been established [26–28]. It brings a fresh view to CNNs. In sparse coding, we assume that a signal can be represented as a linear combination of a few columns from a matrix, called the dictionary, and the linear combination can be written as a sparse vector. The task of retrieving the sparse representation of a signal is called sparse coding or basis pursuit (BP) [29–32]. It is also known in the statistical learning community as the least absolute shrinkage and selection operator (Lasso) problem [33]. Neuroscience also indicates that sparse coding plays an important role in the human brain [34]. Moreover, sparsity has been shown to be a driving force in a myriad of applications in computer vision [35,36] and statistics [33]. For a given dictionary, orthogonal matching pursuit [37,38], the iterative soft thresholding algorithm (ISTA) and its fast version (FISTA) [39] have been proposed to tackle the pursuit problem. Besides, double sparsity has also been proposed to accelerate the training process [40], and it assumes the dictionary can be factorized into a multiplication of two matrices.

Inspired by these advances, a multilayer convolutional sparse coding (ML-CSC) model was proposed by Papyan *et al.* [26], which has been shown to equal a plain network when propagates with the layered thresholding algorithm [26]. This reveals that CNN actually tries to find the sparse coding of input signals over a very special dictionary, which corresponds to a convolution operation. CNNs compute the sparse vectors layer by layer, but does not recover all the vectors at once, which is computationally and conceptually challenging. The value of an ML-CSC model not only gives us an understanding of CNNs, but also builds a strict mathematical form that provides the opportunity of utilizing more mathematical tools to carry out a strict theoretical analysis. Previous studies have explained why ReLU behaves well in numerical experiments [2], what the feature maps computed in each layer represent, and what the meaning of the bias term (which is always added after convolution) is [26].

To solve the CSC model, layered BP and a multilayer version have been proposed [26,28]. Layered BP considers the sparsity in only one layer, while the multilayer version considers the sparsity in all layers. The stability of layered BP in noiseless and noisy regimes was clarified in Ref. [26]. An error bound was proposed to measure the distance between the solution and the true underlying sparse coding [26]. The convergence of the multilayer version was proved in Ref. [28]. The uniqueness of the sparsest representation and the conditions that guarantee finding the true underlying representation were discussed in Ref. [27]. See Ref. [26] for a discussion of other theoretical benefits.

At present, existing studies only establish a preliminary connection between the CSC model and plain networks. The relationship between the CSC model and current popular architectures (e.g. ResNet, MSDNet) is still lacking. The roles of many key tricks (e.g. batch normalization, dropout) in the CSC model are still not clear. We note that neural networks with skip connections usually have better performance [16–18]. Do the skip connections have any theoretical interpretation? Moreover, dilated convolution in the MSDNet is also a powerful trick for extracting multiscale features [18]. To better understand the ResNet and MSDNet, we introduce a residual convolutional sparse coding (Res-CSC) model and a mixed-scale dense convolutional sparse coding (MSD-CSC) model, which are closely related to the ResNet and MSDNet, respectively.

## NOTATION AND CONCEPTS

### Convolution and matrix multiplication

Convolution is a basic operation in CNNs. We denote an image with *m* rows, *n* columns and *c* channels as **X** ∈ *R*^*m* × *n* × *c*^. A dilated convolution kernel *F*^*s*^ with dilation scales *s* ∈ $z$^+^ [41] convolves **X** to produce a new feature map **Z**. This operation is denoted as **Z** = *F*^*s*^⊗**X**. Equally, this process can be written as a matrix multiplication. Simple examples with one channel and different dilation scales *s* = 1 and *s* = 2 are illustrated in Fig. S1 of the online supplementary material. Here, we use the same symbol *F*^*s*^ to represent the corresponding matrix, which has a special structure—a union of bars and circulant. We call *F*^*s*^ the convolutional matrix.

### The ResNet and MSDNet

**Figure 1.**
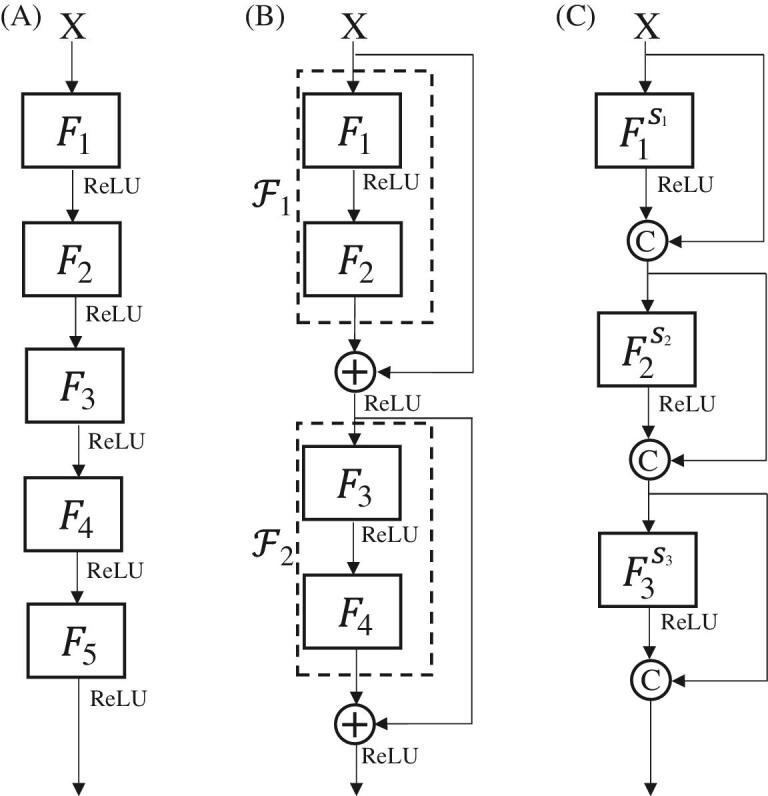
(A) A plain network of five layers formed by simply stacking each layer. (B) A ResNet of two layers. Here we assume that the operation does not change the shape of the tensors. The symbol 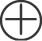 indicates elementwise addition of the tensors in the current and previous layers. (C) An MSDNet of three layers. The symbol 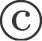 indicates channelwise concatenation of the tensors in the current and previous layers.

Compared with the traditional CNN (Fig. 1A), a ResNet [16] adds one operation, the skip connection (Fig. 1B), and an MSDNet [18] adds two operations, the dilated convolution and dense connection (Fig. 1C). The skip connection directly adds feature maps after a transformation (Fig. 1B). This process can be written as

$$\begin{equation*}
{\bf Z}_{i+1}=\mathcal {F}({\bf Z}_{i})+{\bf Z}_{i},
\end{equation*}$$

where $\mathcal {F}$ is a transformation and **Z**_*i*_ is the *i*th layer output.

Dilated convolutions with different dilation scales could acquire larger receptive fields with fewer parameters, enabling feature extraction in a multiscale manner. Dense connection gathers all feature maps before the current layer and computes new feature maps with them (Fig. 1C). This process can be written as

$$\begin{eqnarray*}
{\bf Z}_{i+1}=\sigma (F_{i+1}^{s_{i+1}}(\lbrace {\bf Z}_{0}, {\bf Z}_{1}, \ldots , {\bf Z}_{i}\rbrace )+b_{i+1}),
\end{eqnarray*}$$

where σ(·) is the ReLU function, **Z**_0_ is an input image, **Z**_*i* + 1_ denotes new feature maps, **Z**_*i*_ is the *i*th layer output, {**Z**_0_, **Z**_1_, $\ldots ,$**Z**_*i*_} denotes feature maps which concatenates **Z**_0_, **Z**_1_, $\ldots ,$**Z**_*i*_, and *b*_*i*+1_ is a bias term.

Here, an MSDNet with *k* layers is represented as

$$\begin{eqnarray*}
{\rm MSD}^{k}&=&\lbrace (F_{1}^{s_{1}}, b_{1}),(F_{2}^{s_{2}}, b_{2}), \ldots ,(F_{k}^{s_{k}}, b_{k})\rbrace ,
\end{eqnarray*}$$

where $(F_{i}^{s_{i}}, b_{i})$ denotes the convolution kernels and the bias *b*_*i*_ in the *i*th layer. Note that *b*_*i*_ is a vector recording biases corresponding to convolution kernels in $F_{i}^{s_{i}}$. Here, we use $w$ to denote the number of convolution kernels in each layer and *d* to denote the number of layers.

### Sparse coding

Let *X* denote a signal vector. In sparse coding, we assume that it can be represented as a linear combination of a few columns of a dictionary matrix *D*:

$$\begin{equation*}
X=D \Gamma .\end{equation*}$$

Here Γ is the coefficient vector of the linear combination, called a coding under the dictionary *D*.

Sparse coding can be formulated as the following optimization problem [29,40,42]:

$$\begin{equation*}
(\mathrm{P}0):\min _{\Gamma } \frac{1}{2}\Vert X-D \Gamma \Vert _{2}^{2}+\beta \Vert \Gamma \Vert _{0}.
\end{equation*}$$

Here β is a regularization parameter to balance the reconstruction error of the signal *X* and the sparsity of Γ. ‖Γ‖_0_ denotes the number of nonzero entries in Γ.

Problem (P0) is NP-hard because of the second term ‖Γ‖_0_ [43]. Fortunately, it has been proved that problem (P0) can be relaxed as Ref. [44]:

$$\begin{equation*}
(\mathrm{P} 1):\min _{\mathrm{\Gamma }} \frac{1}{2}\Vert X-D \Gamma \Vert _{2}^{2}+\beta \Vert \Gamma \Vert _{1}.
\end{equation*}$$

Here problem (P1) is called the Lasso [33] or BP problem [29–32] in different fields. Moreover, problem (P1) can be solved using the popular ISTA. Its update formula is formulated as

(1) \begin{eqnarray*} \Gamma ^{k+1}=S_{{\beta }/{L}}\bigg (\Gamma ^{k}-\frac{1}{L}(-D^\top X+D^\top D \Gamma ^{k})\bigg ), \nonumber\\ \end{eqnarray*}

where Γ^*k*^ denotes the coding in the *k*th iteration. The smallest Lipschitz constant (*L*) of the gradient of $f(\Gamma )=\frac{1}{2}\Vert X-D \Gamma \Vert _{2}^{2}$ is λ_max _(*D*^⊤^*D*), where λ_max _(*D*^⊤^*D*) denotes the maximum eigenvalue of *D*^⊤^*D*. The soft thresholding operator *S*_*b*_(·) is defined as

$$\begin{equation*}
S_{b}(z)=\left\lbrace \begin{array}{@{}l@{\quad }l@{}}z+b, & {z<-b,} \\
{0,} & {-b \le z \le b.} \\
{z-b,} & {z > b.} \end{array}\right.
\end{equation*}$$

Is the sparsest representation for problem (P1) unique? Lemma 1 provides the answer:

**Lemma 1** [29,42,45]. *The sparsest representation is unique if the number of nonzeros in the underlying sparsest representation for problem (P1) is not too high and, in particular, less than $\frac{1}{2}(1+{1}/{\mu (D)})$. Here, μ(*D*) is defined as the maximal inner product of two columns extracted from D. This can be formally written as*

$$\begin{equation*}
\mu (D)=\max _{i \ne j}\left|d_{i}^\top d_{j}\right|,
\end{equation*}$$

*where *d*_*i*_ is assumed to be normalized to the unit length.*

## THE RELATIONSHIP BETWEEN THE CSC MODEL AND CNN

### Non-negative sparse coding

Let us consider a signal *X* = *D*Γ. Naturally, Γ can be split into a positive part, Γ_P_, and a negative part, Γ_N_. Then *X* can be written as

$$\begin{equation*}
X=D \Gamma _{\rm P}+(-D)(-\Gamma _{\rm N}).
\end{equation*}$$

Obviously, if we change the dictionary to [*D*, −*D*] then the corresponding sparse coding is [Γ_P_, −Γ_N_]^⊤^. Note that both Γ_P_ and −Γ_N_ are non-negative. Therefore, every sparse coding can always be converted into non-negative sparse coding [26].

### The soft non-negative thresholding operator

For a non-negative sparse coding problem, we only need to consider the non-negative situation. So, we can define the soft non-negative thresholding operator $S_{b}^{+}(\cdot )$ based on the soft thresholding operator as

$$\begin{equation*}
S_{b}^{+}(z)=\left\lbrace \begin{array}{@{}l@{\quad }l@{}}0, & {z \le b,} \\
{z-b,} & {z > b.} \end{array}\right.
\end{equation*}$$

It is obvious that the soft non-negative thresholding operator is equivalent to the ReLU:

(2) \begin{equation*} S_{b}^{+}(z)=\max (z-b, 0)={\rm ReLU}(z-b), \end{equation*}

where *b* is a bias term. According to Equation (1), *b* depends on β and the Lipschitz constant *L* in problem (P1). In other words, β is a hyperparameter in sparse coding, but it becomes a trainable parameter in neural networks via Equation (2).

### ML-CSC

The ML-CSC model [26] is formulated as

$$\begin{eqnarray*}
X&=&D_{1} \Gamma _{1}, \\
\Gamma _{1}&=&D_{2} \Gamma _{2}, \\
&\vdots &\\
\Gamma _{k-1}&=&D_{k} \Gamma _{k},
\end{eqnarray*}$$

where *X* is the input signal (e.g. an image) and {*D*_*i*_} is a set of special dictionaries. Each *D*_*i*_ is a transpose of a convolutional matrix. Note that we use the equal sign to express reconstruction rather than exact equality. The ML-CSC model encodes signals layer by layer:

$$\begin{eqnarray*}
\begin{array}{c}\Gamma _{1} \text{ reconstructs } X \text{ via dictionary } D_{1}, \\
\Gamma _{2} \text{ reconstructs } \Gamma _{1} \text{ via dictionary } D_{2}, \\
\vdots \\
\Gamma _{k} \text{ reconstructs } \Gamma _{k-1} \text{ via dictionary } D_{k}.
\end{array}
\end{eqnarray*}$$

The *i*th layer in the ML-CSC model can be described as a Lasso problem:

$$\begin{equation*}
(\mathrm{P}2):\min _{\Gamma _{i}} \frac{1}{2}\Vert \Gamma _{i-1}\!-\! D_{i} \Gamma _{i}\Vert _{2}^{2}\!+\!\beta \Vert \Gamma _{i}\Vert _{1}.
\end{equation*}$$

We use Equation (1) to compute the sparse coding in every layer. When {*D*_*i*_} is known, we set Γ^0^ = 0, and Equation (1) becomes

(3) \begin{equation*} \Gamma ^{1}=S_{{\beta }/{L}}\bigg (\frac{1}{L}(D^\top X)\bigg ). \end{equation*}

We only update Γ once with Equation (3) and then obtain the layered thresholding algorithm [26] (see Algorithm 1 in the online supplementary material).

According to the relationship between convolution and matrix multiplication, it is obvious that using this layered thresholding algorithm to solve the ML-CSC model is equivalent to the forward pass of plain networks (Fig. 1A) [26]. So, the final sparse coding Γ_*k*_ in the ML-CSC model corresponds to the final feature map in the CNN. Here we set Γ^0^ = 0 in each layer and only update Γ once. This strategy not only achieves the equivalence between the ML-CSC model and plain networks, but also improves the computational efficiency since two terms, Γ^*k*^ and *D*^⊤^*D*Γ^*k*^, are ignored. A different initialization will be discussed in the next section.

Until now, the ML-CSC model has been connected to plain networks. Intriguingly, we believe that three factors in each layer, the initialization, the dictionary design and the number of iterations, greatly affect its performance. Inspired by these considerations, in the next three sections we propose the Res-CSC and MSD-CSC models and a forward propagation algorithm with unfolding (iterate more than once), respectively.

## LAYER-INITIALIZING QUESTION AND THE RES-CSC MODEL

According to the above analysis, the forward pass of plain networks can be explained by solving (P1) with initialization Γ^0^ = 0 in each layer. It dramatically improves the computational efficiency. However, this naive setting might cause large errors because the information from the other two terms is ignored. The errors are accumulated layer by layer. It leads to training difficulty in much deeper networks. Thus, we propose a fundamental question about initialization.

**Layer-Initializing Question.** In the ML-CSC model, Equation (1) iterates once in each layer. Under this condition, can we design a proper initialization for $\Gamma _i^{0}$ in the *i*th layer to approach the optimal sparse coding Γ_*i*_?

To give a solution to the layer-initializing question (LIQ), we modify the ML-CSC model. Intuitively, according to the form of the soft thresholding operator, large values in the coding will be decreased by a constant, and small values become zeros directly. This results in the coding values moving towards zero. In the view of ML-CSC, the forward pass of CNN is a series of sparse coding problem. It repeats the soft thresholding operator layer by layer. The coding values of each layer move towards zero again and again. Thus, the coding gradually becomes sparse. An intuitive idea is to set Γ^0^ equal to the input of a former layer. In the following, we use the input of the layer closest to the current layer as the initialization (denoted as Γ^0^ = *X*_−1_). We change the forward propagation rule of each layer partially in the ML-CSC model to reduce the accumulation error, and keep part of the rule to enhance the computational efficiency. Specifically, every two layers we use this new initialized setting once. In the first layer, we adopt Equation (3) to obtain the output, and in the second layer we employ the following update rule based on Equation (1):

(4) \begin{eqnarray*} \Gamma ^{1}=S_{{\beta }/{L}}\bigg (\frac{1}{L} D^\top X+X_{-1}-\frac{1}{L} D^\top D X_{-1}\bigg ). \nonumber\\ \end{eqnarray*}

Let $\mathfrak {D}={D}/{L}$ and *c* = −*L*. Equation (4) becomes

(5) \begin{eqnarray*} \Gamma ^{1}=S_{{\beta }/{L}}(\mathfrak {D}^\top X+X_{-1}+\mathbf {c} \cdot \mathfrak {D}^\top \mathfrak {D} X_{-1}). \nonumber\\ \end{eqnarray*}

**Figure 2.**
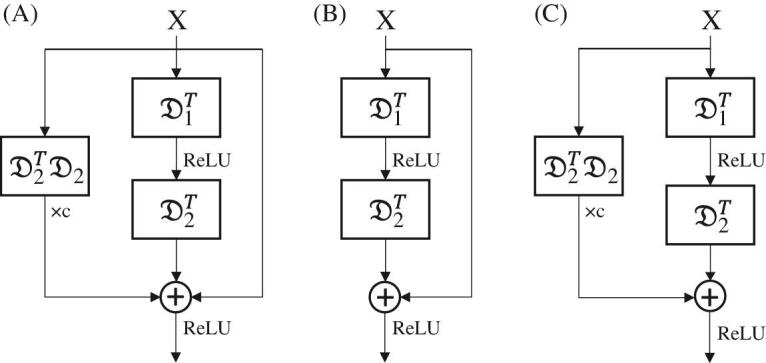
(A) A component of the Res-CSC model with two continuous layers. (B) A component of the ResNet with two continuous layers, which can be seen as a simplification of (A). (C) Another way to simplify (A) (referred to as the Res-CSC-simplified model). Here, $\mathfrak {D_1}$ and $\mathfrak {D_2}$ are the dictionaries of the two continuous layers.

Now we obtain a new optimization rule following the same mode of ML-CSC. In contrast, its forward propagation implements Equations (3) and (5) alternately (Fig. 2A). Note that Equations (5) becomes the forward propagation of the ResNet exactly when we ignore the term $\mathbf {c} \cdot \mathfrak {D}^\top \mathfrak {D} X_{-1}$. For convenience, we call the ML-CSC model updated with this new rule the Res-CSC model. The classical ResNet can be seen as a special case of the Res-CSC model (Fig. 2B), which gives an approximate solution to the LIQ. However, it has not been formally proposed before that the LIQ is a key of the optimization problem behind the degradation phenomenon [16]. Coincidentally, the ResNet addresses it by introducing such nonzero initializations. Similarly, we can obtain another approximate update rule by ignoring the term *X*_−1_ (Fig. 2C).

The relationship between the Res-CSC model and ResNet draws our attention to the concept of error tolerance, which is an important characteristic of the CNN  [46,47]. Being the approximation of the Res-CSC model, the ResNet presents this characteristic in many applications. For a more general propagation rule, the Res-CSC model is expected to overcome the training difficulty when networks comprise hundreds of layers. In the Experiments section, numerical tests on the Res-CSC model, ResNet and Res-CSC-simplified model indeed demonstrate that the Res-CSC model could achieve this and the term $\mathbf {c} \cdot \mathfrak {D}^\top \mathfrak {D} X_{-1}$ in Equation (5) plays a slight role.

## THE MSD-CSC MODEL AND THEORETICAL ANALYSIS

### The MSD-CSC model

Inspired by the layered thresholding algorithm designed for the ML-CSC model, we attempt to describe the dilated convolution and dense connection of the MSDNet from the view of sparse coding by modifying the structure of dictionaries. In the MSDNet, each layer uses all the previous feature maps to compute its layer output. This leads to the CSC model:

$$\begin{eqnarray*}
X&=& D_{1}^{s_{1}} \Gamma _{1}, \\
\Gamma _{1}&=& D_{2}^{s_{2}} \Gamma _{2}, \\
&\vdots &\\
\Gamma _{k-1}&=& D_{k}^{s_{k}} \Gamma _{k},
\end{eqnarray*}$$

where *X* is the input signal (e.g. an image), $D_{i}^{s_{i}}=[{{I}}, {(F_{i}^{s_{i}})^\top }]$ and *I* is an identity matrix. The Lasso problem in the *i*th layer is formulated as

$$\begin{equation*}
\min _{\Gamma _{i}} \frac{1}{2}\Vert \Gamma _{i-1}-D_{i}^{s_{i}} \Gamma _{i}\Vert _{2}^{2}+\beta \Vert \Gamma _{i}\Vert _{1}.
\end{equation*}$$

We call this the MSD-CSC model and denote it as

$$\begin{eqnarray*}
{\rm MSDCSC}^{k} = \lbrace (D_{1}^{s_{1}}, \beta _{1}),(D_{2}^{s_{2}}, \beta _{2}), \ldots ,(D_{k}^{s_{k}}, \beta _{k})\rbrace ,
\end{eqnarray*}$$

where $D_{i}^{s_{i}}$ is the dictionary and β_*i*_ is the regularization parameter in the *i*th layer.

### Theoretical analysis

**Proposition 1.** *For a given MSDNet, there exists an MSD-CSC model that is equivalent to the MSDNet when propagates with the layered thresholding algorithm.*

*Proof*. For a given MSDNet,

$$\begin{eqnarray*}
{\rm MSD}^{k}&=&\lbrace (F_{1}^{s_{1}}, b_{1}),(F_{2}^{s_{2}}, b_{2}),\ldots ,(F_{k}^{s_{k}}, b_{k})\rbrace .
\end{eqnarray*}$$

Let us define the corresponding MSD-CSC model

$$\begin{eqnarray*}
{\rm MSDCSC}^{k}=\lbrace (D_{1}^{s_{1}}, \beta _{1}),(D_{2}^{s_{2}}, \beta _{2}), \ldots ,(D_{k}^{s_{k}}, \beta _{k})\rbrace ,
\end{eqnarray*}}$$

where β_*i*_ = (0, $\ldots ,$ 0, *L*_*i*_*b*_*i*_)^⊤^ with *L*_*i*_ the Lipschitz constant in the *i*th layer. According to the layered thresholding algorithm

$$\begin{eqnarray*}
\widehat{\Gamma }_{i} &=& S^+_{\overline{b}_{i}}((D_{i}^{s_{i}})^\top \hat{\Gamma }_{i-1}) \\
&=& S^+_{\overline{b}_{i}}\left( \left[{\begin{array}{c}I \\
{F_{i}^{s_{i}}} \end{array}} \right]\hat{\Gamma }_{i-1}\right) \\
&=& S^+_{\overline{b}_{i}}\left(\left[ {\begin{array}{c}\hat{\Gamma }_{i-1} \\
{F_{i}^{s_{i}}\widehat{\Gamma }_{i-1}} \end{array}}\right] \right) \\
&=& \text{ReLU}\left(\left[ {\begin{array}{c}\hat{\Gamma }_{i-1} \\
{F_{i}^{s_{i}}\widehat{\Gamma }_{i-1}+b_{i}} \end{array}}\right] \right) \\
&=& \left[{\begin{array}{c}\hat{\Gamma }_{i-1} \\
{Z_{i}} \end{array}}\right] \\
&=&\text{concatenate}(\hat{\Gamma }_{i-1}, Z_{i}),
\end{eqnarray*}$$

where *i* = 1, $\ldots ,$*k* and $\overline{b}_{i}={\beta _{i}}/{L_{i}}=(0,\ldots ,0,b_{i})$. We observe that the features before the *i*th layer are kept in the *i*th feature as the input of the next layer. This indicates that the propagating rule of MSDCSC^*k*^ is equivalent to that of MSD^*k*^.

From **Proposition**1, we can see that the identity matrix corresponds to the dense connection in neural networks. Consider the process of reconstructing the original signal in the MSD-CSC model. We reconstruct $\hat{\Gamma }_{i-1}$ using

$$\begin{eqnarray*}
\left[{{I}}, {(F_{i}^{s_{i}})^\top }\right] \left[{\begin{array}{c}\hat{\Gamma }_{i-1} \\
{Z_{i}} \end{array}}\right] =\hat{\Gamma }_{i-1}+{(F_{i}^{s_{i}})^\top {Z_{i}}}.
\end{eqnarray*}$$

Here ${(F_{i}^{s_{i}})^\top {Z_{i}}}$ is a residual term to reconstruct the signal in the previous layer, while the residual term in the ResNet produces coding in the next layer.

Next, we prove that the coding performance of the MSD-CSC model is better than that of the ML-CSC model. This is due to two operations: dilation convolution and dense connection. In the context of the CSC model, the dilation convolution affects the structure of the convolutional matrices. The μ(*D*) is relatively smaller in the convolutional matrix, corresponding to the dilated convolutional kernel compared with that without dilation (see Figs S1B and S1C in the online supplementary material for μ = 0.47 and μ = 0, respectively). According to **Lemma 1**, a dictionary (note that the identity matrices in the MSD-CSC model do not affect μ) with smaller μ tends to ensure that the sparsest representation is unique. More theoretical studies on uniqueness and stability relating to μ(*D*) are analyzed in Ref. [26].

We now explore how the MSD-CSC model benefits from the dense connection. According to Lasso, larger β leads to sparser representation, but sparser representation may cause the loss of information. Sparsity and loss of information are contradictory. Sometimes, an unsuitable β can lead to a very unreasonable solution. For example, set

$$\begin{eqnarray*}
D=\left[{\begin{array}{ccc}0.1 &\quad {0.1} &\quad {0.2} \\
{0.1} &\quad {0.2} &\quad {0.1} \end{array}}\right] \quad \text{and}\quad \beta =\frac{1}{3}.
\end{eqnarray*}$$

According to Equation (1), the coding of the signal *X* = (1, 1)^⊤^ over *D* is Γ = (0, 0, 0)^⊤^. It is obviously unreasonable that all the information is lost. This ill-conditioned case leads to the following definition.

**Definition 1.** For the encoding process in the *i*th layer, $\Gamma _{i-1}=D_{i}^{s_{i}} \Gamma _{i}$, the corresponding Lasso problem is

$$\begin{equation*}
\min _{\Gamma _{i}} \frac{1}{2}\Vert \Gamma _{i-1}-D_{i}^{s_{i}} \Gamma _{i}\Vert _{2}^{2}+\beta \Vert \Gamma _{i}\Vert _{1}.
\end{equation*}$$

Let $\xi =(\xi _{1}, \xi _{2}, \ldots , \xi _{n})=D_{i}^{s_{i}} \Gamma _{i}$, which is used to reconstruct the signal Γ_*i* − 1_. If the *j*th dimension satisfies

$$\begin{equation*}
|\xi _{j}-\Gamma _{i-1}^{(j)}|>2 \beta ,
\end{equation*}$$

where $\Gamma _{i-1}^{(j)}$ denotes the value in the *j*th dimension of Γ_*i* − 1_, the reconstruction is considered unsuccessful in the *j*th dimension; otherwise, it is considered a success.

**Figure 3.**
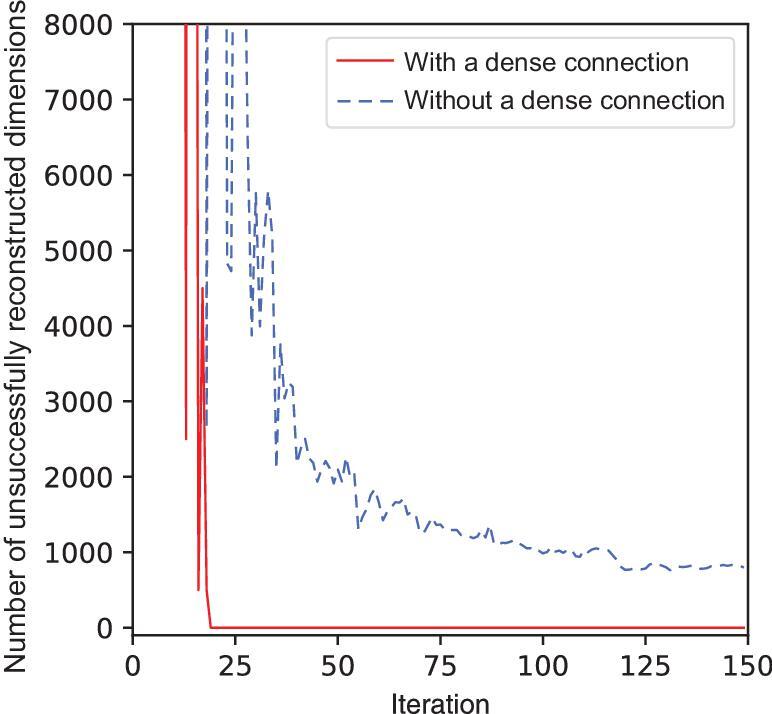
The number of unsuccessfully reconstructed dimensions in the original signal after each iteration identified by the models with (MSD-CSC) or without (ML-CSC) the dense connection.

Let us conduct a simulated experiment on the ML-CSC model first. The simulation data is of length 100 and generated by adding the Gaussian noise to 100 different centers, which represent 100 different classes. We generate 10 000 training data (each class has 100 data points) and 2000 testing data. We use an ML-CSC model with two hidden layers. After each training iteration, we compute the reconstruction of the input signal *X* and count the number of dimensions that fail to be reconstructed (i.e. unsuccessful) (see the dotted line in Fig. 3). We can see that the number of unsuccessfully reconstructed dimensions decreases at the beginning of the training process and becomes stable after some iterations. Finally, there still exist some dimensions that cannot be reconstructed successfully. Similarly, we repeat the simulated experiment on the MSD-CSC model. We can see that the number of unsuccessfully reconstructed dimensions decreases rapidly compared with that in the ML-CSC model and finally stabilizes at zero (see the solid line in Fig. 3). This means that the unsuccessfully reconstructed dimensions in the ML-CSC model can be recovered in the MSD-CSC model. We can prove this phenomenon in theory as follows (see the online supplementary material for the proof).

**Theorem 1.** *In the MSD-CSC model, all dimensions in Γ_*i* − 1_ are reconstructed successfully.*

According to the proof of **Theorem 1**, the identity matrix in the dictionary is critical. It prevents the loss of information and makes the reconstructed value not so far from the original value. Besides, the identity matrix brings an additional benefit. The MSD-CSC model allows larger βs than the ML-CSC model with the same parameters in convolution kernels based on the following lemma (see the online supplementary material for the proof).

**Lemma 2.** *For a matrix* *A* ≠ 0, *assume that the matrix *AA*^⊤^ has eigenvalues λ*_1_, $\ldots ,$*λ_*n*_. As a result, the matrix*

$$\begin{equation*}
B = \left({\begin{array}{c}I \\
{A} \end{array}}\right) \cdot ({I}, {A^\top })= \left({\begin{array}{cc}I &\quad {A^\top } \\
{A} &\quad {A A^\top } \end{array}}\right)
\end{equation*}$$

*has eigenvalues* 0, $\ldots ,$ 0, *λ*_1_ + 1, $\ldots ,$*λ_*n*_* + 1. *Here the number of zeros is equal to the column number of A.*

In the MSD-CSC model, the Lipschitz constant

$$\begin{equation*}
L_{\text{MSD-CSC}}\!=\!\lambda _{\max }\left(\! \left(\begin{array}{c}I \\
{F_{i}^{s_{i}}} \end{array}\right) ({I}, {(F_{i}^{s_{i}})^\top })\! \right).
\end{equation*}$$

According to **Lemma 2**, *L*_MSD-CSC_ = *L*_ML-CSC_ + 1, where *L*_ML-CSC_ is the Lipschitz constant of the corresponding ML-CSC model. According to Equations (1) and (2), β = *bL*. So

$$\begin{eqnarray*}
\beta _{\text{MSD-CSC}}&=& b(L_{\text{ML-CSC}}+1)\nonumber\\
&=&\beta _{\text{ML-CSC}}+b.
\end{eqnarray*}$$

Clearly, the β in the MSD-CSC model becomes larger than that in the corresponding ML-CSC model. This means that the MSD-CSC model tends to obtain sparser solutions. In addition, according to the proof of **Theorem 1**, the MSD-CSC model can prevent loss of information via the identity matrix in the dictionary. Thus, the MSD-CSC model alleviates the contradiction between reconstruction and sparsity. Besides, the identity matrix corresponds to the dense connection in the MSDNet according to **Proposition 1**. Taken together, **Proposition 1** and **Theorem 1** provide a complete theoretical understanding of the dense connection in neural networks. Finally, we summarize the relationship between the generalized CNNs (including the ResNet and MSDNet) and the new CSC models in Table 1.

**Table 1.** The relationship between the generalized CNN and generalized CSC model.

CNN	CSC
The *i*th convolution with dilation scale *s*_*i*_	The convolutional matrix $D_{i}^{s_{i}}$
Bias term	The balance coefficient β and λ_max _(*D*^⊤^*D*)
ReLU	Soft non-negative thresholding operator $S_{\beta }^{+}(\cdot )$
Feed-forward algorithm	Γ^0^ = 0 in the update formula and iterate once
ResNet	Γ^0^ = *X*_−1_ in the update formula and iterate
	Equations (3) and (5) alternately
Dense connection	The identity matrix in $D_{i}^{s_{i}}$

## FORWARD PROPAGATION ALGORITHM

For the MSD-CSC model, we just need to replace the dictionary in Equation (1) with the corresponding dictionary. The update formula becomes

$$\begin{eqnarray*}
\Gamma ^{k+1}&=& S_{{\beta }/{L}}\bigg(\Gamma ^{k}-\frac{1}{L} \left[{\begin{array}{c}I \\
{F_{i}^{s_{i}}} \end{array}}\right]\nonumber\\
&& \times(-\mathrm{X}+[{I}, {(F_{i}^{s_{i}})^\top }] \Gamma ^{k})\bigg).
\end{eqnarray*}$$

We can adopt the ISTA and FISTA [39] to tackle problem (P1). Besides, it is unnecessary to limit the number of iterations to one. Iterating more than once corresponds to the unfolding operation, which has also been explored for solving sparse coding and other problems. Here we adopt it to improve the performance of the dense connection in the MSDNet. The case of unfolding =0 corresponds to the MSDNet. The models with different unfolding have the same number of parameters (see Fig. S3A in the online supplementary material). According to the relationship between convolution and matrix multiplication, we can obtain the forward propagation algorithm in one layer (see Algorithms 2 and 3 in the online supplementary material).

The main difference between the two algorithms is that the ISTA is based only on the last iteration, but the FISTA is based on a linear combination of the last two iterations. Obviously, the main computational effort in both algorithms remains the same. We illustrate a simple architecture using the MSD-CSC model for a classification task (see Fig. S3B in the online supplementary material). In this architecture, each block represents a layer in the MSD-CSC model. We can implement a propagating algorithm and set an unfolding number (e.g. 0, 1, 2) in each block. Note that the FISTA is the same as the ISTA when unfolding <2. How the feature maps in each block propagate is illustrated in Fig. S3A of the online supplementary material. Max-pooling layers are added to downsample feature maps for memory constraints. Obviously, the number of blocks corresponds to *d* and the number of convolution kernels corresponds to $w$ in the MSDNet.

## EXPERIMENTS

In this section, we evaluate the Res-CSC and MSD-CSC models and related methods using the three typical datasets CIFAR10, CIFAR100 and SVHN [48,49]. CIFAR10, CIFAR100 and SVHN consist of colored natural images with 32 × 32 pixels. In SVHN, the training and testing sets contain 73 257 images and 26 032 images, respectively. In CIFAR10 and CIFAR100, the training and testing sets contain 50 000 and 10 000 images, which are drawn from 10 and 100 classes, respectively.

### Experiments on the Res-CSC model

We implement standard data augmentation (translation and horizontal flip) on CIFAR10 and CIFAR100. The four models, the plain network, Res-CSC-simplified, ResNet and Res-CSC, are trained with the stochastic gradient descent (SGD) on a single GPU. The mini-batch size is 128 and the Nesterov momentum is set to 0.9. Each model is trained for 200 epochs. The initial learning rate is set to 0.1 and then divided by 10 after 100 and 150 epochs.

**Figure 4.**
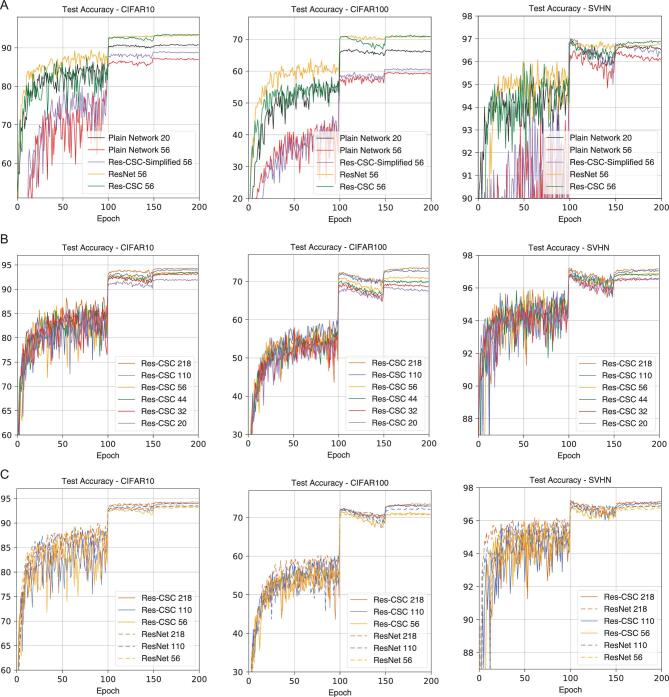
Experiments on the Res-CSC model and other methods using SVHN, CIFAR10 and CIFAR100. (A) Test accuracy versus the training epochs of the plain network Res-CSC-simplified, ResNet and Res-CSC with 56 layers and the plain network with 20 layers for comparison. (B) Test accuracy versus the training epochs of the Res-CSC model with 20, 32, 44, 56, 110 and 218 layers. (C) Test accuracy versus the training epochs of the Res-CSC model and ResNet with 56, 110 and 218 layers.

First, we train the four types of networks with 20 and 56 layers, respectively. The plain network with 56 layers achieves lower accuracy than that with 20 layers (Fig. 4A and Table 2), suggesting that the degradation phenomenon occurs. Interestingly, the degradation phenomenon is alleviated to some extent with the Res-CSC-simplified model though the improvement is limited. Moreover, when the number of layers is on the order of hundreds, the Res-CSC-simplified model cannot be trained normally, as in the case of the plain network. Both the Res-CSC and ResNet distinctly overcome the degradation phenomenon (Fig. 4B and C). These observations are consistent with our theoretical derivation. According to the results, the term *X*_−1_ plays a more important role than the term $\mathbf {c} \cdot \mathfrak {D}^\top \mathfrak {D} X_{-1}$.

**Table 2.** The accuracy rates of a plain network, Res-CSC-simplified, ResNet and Res-CSC on CIFAR10, CIFAR100 and SVHN.

			Model^*a*^			
Dataset	Layer	Parameter	Plain network	Res-CSC-simplified	ResNet	Res-CSC
			(%)	(%)	(%)	(%)
CIFAR10	20	0.27M*^b^*	90.74	**91.86**	91.30	91.82
	32	0.46M	90.34	90.95	92.52	**93.31**
	44	0.66M	89.41	90.19	**92.92**	92.89
	56	0.85M	87.04	88.75	93.15	**93.55**
	110	1.70M	×*^c^*	×	93.41	**93.56**
	218	3.40M	×	×	94.02	**94.28**
CIFAR100	20	0.27M	66.02	66.33	67.81	**68.55**
	32	0.46M	64.31	65.35	**69.79**	69.35
	44	0.66M	61.54	63.24	70.59	**70.68**
	56	0.85M	59.28	60.58	71.09	**71.40**
	110	1.70M	×	×	72.47	**73.13**
	218	3.40M	×	×	73.28	**73.50**
SVHN	20	0.27M	96.67	96.70	96.57	**96.81**
	32	0.46M	96.55	96.63	96.63	**96.83**
	44	0.66M	96.36	96.51	96.74	**96.91**
	56	0.85M	96.09	96.38	96.88	**97.01**
	110	1.70M	×	×	96.96	**97.11**
	218	3.40M	×	×	97.05	**97.22**

^*a*^ The top result of the four methods in each setting is shown in boldface. ^*b*^ The number of trainable parameters is counted in millions (M). ^*c*^ × indicates the model can not be trained normally.

Next, we train six Res-CSC and ResNet models with 20, 32, 44, 56, 110 and 218 layers, respectively. Each Res-CSC model has the same number of parameters as the corresponding ResNet. Clearly, the Res-CSC model achieves very competitive or even slightly better performance than the ResNet in terms of accuracy (Fig. 4 and Table 2). The difference is due to the effect of the last term in Equation (5). Besides, the Res-CSC model takes a little more time for extra convolution and transposed convolution (see the online supplementary material). In short, the Res-CSC model is a more general white-box model for overcoming the degradation phenomenon. Its special case with *c* = 0 leads to an equivalent form of the ResNet. Thus, it can be an alternative to the black-box ResNet. More importantly, it leads to a more theoretical understanding of the ResNet in terms of the update rule.

### Experiments on the MSD-CSC model

We implement MSDNet architectures with $w$ = 32, *d* = 6, 9, 12, *s* = 1, 2, 3 and use max-pooling layers after one-third and two-thirds of the whole layers, respectively (see Fig. S3B in the online supplementary material). Before the softmax layer, average pooling is applied. We compare the results of the ISTA and FISTA with unfoldings of 0, 1 and 2. In addition, we choose the traditional feed-forward network (baseline) and ML-CSC model (six layers, with kernel sizes 4 × 4, 4 × 4, 4 × 4, 3 × 3, 3 × 3 and 3 × 3, respectively, a stride of 2 in the first three layers and a stride of 1 in the last three layers). The number of kernels in each layer is set to 32, 64, 128, 256, 512 and 512 for comparison. These models are trained with SGD on a single GPU with momentum 0.9. The total training epoch is set to 150. The mini-batch size is 128. The initial learning rate is set to 0.05 and then divided by 10 after 75 and 115 epochs.

**Figure 5.**
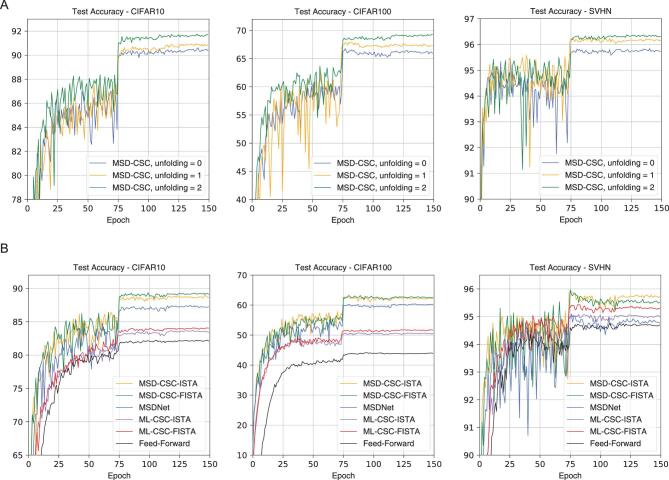
Experiments on the MSD-CSC model and other methods using SVHN, CIFAR10 and CIFAR100. (A) Test accuracy versus the training epochs of the MSD-CSC model with *d* = 12 layers and unfoldings of 0, 1, 2 using the FISTA. (B) Test accuracy versus the training epochs of the MSD-CSC model with *d* = 6 layers, unfolding =2 and other methods.

First, we can clearly see that further unfolding improves the accuracy compared to the MSDNet without unfolding implicitly (Fig. 5A and Table 3). The MSD-CSC model with *d* = 12 and unfolding =2 improves 1.28% and 3.05% on CIFAR10 and CIFAR100, respectively, compared with the corresponding MSDNet. It should be emphasized that this improvement is achieved without adding any extra parameters. Second, the MSD-CSC model uses fewer parameters, but achieves more accurate results compared to other models (Table 3 and Fig. 5B). That the MSD-CSC model has a fewer number of parameters indicates that it has better coding ability. This is consistent with our theoretical analysis. Note that the MSD-CSC model takes more time to train though it has fewer parameters (see the online supplementary material). The reason is that existing deep learning training software does not support the dilation convolution and dense connection operations well since they assume that all channels of a certain feature map are computed in the same way, and GPU convolution routines such as the cuDNN library assume that feature data is stored in a contiguous memory. Therefore, concatenate operation can be expensive in the current software [50]. Frequent concatenate and split operations are used in the MSD-CSC model (see Fig. S3A in the online supplementary material). This limits the application of the MSD-CSC model with more unfolding and layers. We believe that such an implementation issue could be addressed with improved software in the near future.

**Table 3.** The accuracy rates of the MSD-CSC model and other classic CSC models on CIFAR10, CIFAR100 and SVHN.

				CIFAR10	CIFAR100	SVHN
Model^*a*^	Layer	Parameter	Unfolding	(%)	(%)	(%)
Feed-Forward	6	4.0M*^b^*	0	82.16	43.93	94.68
ML-CSC-ISTA	6	4.0M	1	83.26	50.23	94.98
		4.0M	2	83.59	50.61	95.01
ML-CSC-FISTA	6	4.0M	2	84.06	51.66	95.29
MSDNet	6	0.1M	0	87.22	60.23	94.72
	9	0.3M	0	89.42	63.22	95.42
	12	0.6M	0	90.35	66.18	95.73
MSD-CSC-ISTA	6	0.1M	1	88.79	62.08	94.87
			2	88.85	62.24	95.75
	9	0.3M	1	90.23	64.15	95.98
			2	90.52	65.21	96.10
	12	0.6M	1	90.81	67.31	96.14
			2	**91.76**	**68.14**	**96.36**
MSD-CSC-FISTA	6	0.1M	2	89.21	62.53	95.50
	9	0.3M	2	90.91	65.22	95.84
	12	0.6M	2	**91.63**	**69.23**	**96.35**

^a^Under the same setting, the MSDNet corresponds to MSD-CSC-ISTA with unfolding =0 and the FISTA is the same as the ISTA when unfolding <2. The top two cases in each dataset are shown in boldface. *^b^* The number of trainable parameters is counted in millions (M).

## CONCLUSION

Inspired by the relationship between neural networks and the ML-CSC model, we develop the Res-CSC model to explore the LIQ. Intriguingly, the ResNet can be seen as a special case of the Res-CSC model. Hence, we think the LIQ is the key issue behind the degradation phenomenon, which has not been formally proposed before. Through evaluation on three common datasets, we find that the Res-CSC model achieves very competitive or even slightly better performance compared to that of the ResNet. Next, we introduce the MSD-CSC model to decipher the emerging MSDNet architecture via adapting the dictionaries in the ML-CSC model. Through the analysis of this model, we give a theoretical understanding of the dilated convolution (mixed scale) and dense connection in the MSDNet. As we know that sparse coding has more complete theory compared with neural networks. Thus, the bridge between sparse coding and neural networks makes it possible to interpret advanced neural networks. In addition, sparse coding models can be implemented and solved with elegant mathematical optimization algorithms, such as the ISTA and FISTA. Numerical experiments show that the MSD-CSC model performs better than the ML-CSC model because of the advantage of the MSDNet. Moreover, it also performs better than the MSDNet because of the power of the ISTA and FISTA with the unfolding trick, which achieve distinctive improvements without extra parameters.

We conclude some further thinking and potential research directions. First, as shown in this paper, the Res-CSC model gives an answer to the LIQ. Is this answer the best? We think it is a challenge to find a universal rule for finding the best initialization since we would meet different features in different layers. Meta learning may give a potential solution through drawing lessons to learn the initializations. Second, we can see that dilated convolution corresponds to the dilated convolutional dictionary, and the dense connection corresponds to an identity matrix in the dictionary. Can we find a better dictionary structure inspired by such observations? And what operation does this new dictionary structure correspond to? This would help us find a new basic operation used for extracting features from data.

Since the Res-CSC and MSD-CSC models both belong to the ML-CSC model, the error bound for the ML-CSC model [26] also applies to the Res-CSC and MSD-CSC models. The question is whether there exists a tighter bound, which we expect to answer in future work. Moreover, some architectures or operations in neural networks still lack theoretical understanding (e.g. batch normalization, dropout). Can we explain them in a sparse coding framework? On the one hand, we expect to find a mathematical understanding and improve the original models. On the other hand, we hope to find the key roles that these models play in the context of sparse coding.

## Supplementary Material

nwaa159_Supplemental_File

## References

[1] Rosenblatt F . The perceptron: a probabilistic model for information storage and organization in the brain. Psychol Rev 1958; 65: 386–408. 13602029 10.1037/h0042519

[2] Glorot X , Bordes A, Bengio Y. Deep sparse rectifier neuralnetworks. In: Proceedings of the 14th International Conference on Artificial Intelligence and Statistics, 2011, 315–23.

[3] Hubel DH , Wiesel TN. Receptive fields of single neurones in the cat’s striate cortex. J Physiol 1959; 148: 574–91. 14403679 10.1113/jphysiol.1959.sp006308 PMC1363130

[4] Fukushima K . A self-organizing neural network model for a mechanism of pattern recognition unaffected by shift in position. Biol Cybern 1980; 36: 193–202. 7370364 10.1007/BF00344251

[5] Waibel AH , Hanazawa T, Hinton GE et al. Phoneme recognition using time-delay neural networks. IEEE Trans Acoust Speech Signal Process 1989; 37: 328–39.

[6] LeCun Y , Boser BE, Denker JS et al. Backpropagation applied to handwritten zip code recognition. Neural Comput 1989; 1: 541–51.

[7] Klöckner A , Pinto N, Lee Y et al. PyCUDA and PyOpenCL: a scripting-based approach to GPU run-time code generation. Parallel Comput 2012; 38: 157–74.

[8] Abadi M , Barham P, Chen J et al. Tensorflow: a system for large-scale machine learning. In: Proceedings of the 12th USENIX Symposium on Operating Systems Design and Implementation, 2016, 265–83.

[9] Jia Y , Shelhamer E, Donahue J et al. Caffe: convolutional architecture for fast feature embedding. In: Proceedings of the 22nd ACM International Conference on Multimedia, 2014, 675–8.

[10] Krizhevsky A , Sutskever I, Hinton GE. Imagenet classication with deep convolutional neural networks. In: Advances in Neural Information Processing Systems, 2012, 1097–105.

[11] Simonyan K , Zisserman A. Very deep convolutional networks for large-scale image recognition. arXiv:1409.1556

[12] Bengio Y , Simard P, Frasconi P. Learning long-term dependencie with gradient descent is difficult. IEEE Trans Neural Networks 1994; 5: 157–66. 18267787 10.1109/72.279181

[13] Glorot X , Bengio Y. Understanding the difficulty of training deep feedforward neural networks. In: Proceedings of the 13th International Conference on Artificial Intelligence and Statistics, 2010, 249–56.

[14] He K , Zhang X, Ren S et al. Delving deep into rectifiers: surpassing human-level performance on imagenet classiffication. In: Proceedings of the IEEE International Conference on Computer Vision, 2015, 1026–34.

[15] Ioffe S , Szegedy C. Batch normalization: accelerating deep network training by reducing internal covariate shift. arXiv:1502.03167.

[16] He K , Zhang X, Ren S et al. Deep residual learning for image recognition. In: Proceedings of the IEEE Conference on Computer Vision and Pattern Recognition, 2016, 770–8.

[17] Li CY , Vu NT. Densely connected convolutional networks for speech recognition. In: Proceedings of ITG-Symposium on Speech Communication, 2018, 1–5.

[18] Pelt DM , Sethian JA. A mixed-scale dense convolutional neural network for image analysis. Proc Natl Acad Sci USA 2018; 115: 254–9. 29279403 10.1073/pnas.1715832114 PMC5777062

[19] Srivastava N , Hinton G, Krizhevsky A et al. Dropout: a simple way to prevent neural networks from overfitting. J Mach Learn Res 2014; 15: 1929–58.

[20] Szegedy C , Liu W, Jia Y et al. Going deeper with convolutions. In: Proceedings of the IEEE Conference on Computer Vision and Pattern Recognition, 2015, 1–9.

[21] Girshick R , Donahue J, Darrell T et al. Rich feature hierarchies for accurate object detection and semantic segmentation. In: Proceedings of the IEEE Conference on Computer Vision and Pattern Recognition, 2014, 580–7.

[22] Redmon J , Divvala S, Girshick R et al. You only look once Unified, real-time object detection. In: Proceedings of the IEEE Conference on Computer Vision and Pattern Recognition, 2016, 779–88.

[23] Long J , Shelhamer E, Darrell T. Fully convolutional networks for semantic segmentation. In: Proceedings of the IEEE Conference on Computer Vision and Pattern Recognition, 2015, 3431–40. 10.1109/TPAMI.2016.2572683 27244717

[24] Bristow H , Eriksson AP, Lucey S. Fast convolutional sparse coding. In: Proceedings of the IEEE Conference on Computer Vision and Pattern Recognition, 2013, 391–8.

[25] Wohlberg B . Efficient convolutional sparse coding. In: Proceedings of the IEEE International Conference on Acoustics, Speech and Signal Processing (ICASSP), 2014, 7173–7.

[26] Papyan V , Romano Y, Elad M. Convolutional neural networks analyzed via convolutional sparse coding. J Mach Learn Res 2017; 18: 2887–938.

[27] Papyan V , Sulam J, Elad M. Working locally thinking globally: Theoretical guarantees for convolutional sparse coding. IEEE Trans Signal Process 2017; 65: 5687–701.

[28] Sulam J , Aberdam A, Beck A et al. On multi-layer basis pursuit, efficient algorithms and convolutional neural networks. IEEE Trans Pattern Anal Mach Intell 2020; 42: 1968–80. 30869611 10.1109/TPAMI.2019.2904255

[29] Donoho DL , Elad M. Optimally sparse representation in general (nonorthogonal) dictionaries via L1 minimization. Proc Natl Acad Sci USA 2003; 100: 2197–202. 16576749 10.1073/pnas.0437847100 PMC153464

[30] Chen SS , Donoho DL, Saunders MA. Atomic Decompositio by Basis Pursuit. SIAM J Sci Comput 1998; 20: 33–61.

[31] Chen SS , Donoho DL, Saunders MA. Atomic decompositio by basis pursuit. SIAM Rev 2001; 43: 129–59.

[32] Tropp JA. Just relax: convex programming methods for identifying sparse signals in noise. IEEE Trans Inf Theory 2006; 52: 1030–51.

[33] Tibshirani R. Regression shrinkage and selection via the lasso. J R Stat Soc Series B Stat Methodol 1996; 58: 267–88.

[34] Spanne A , Jörntell H. Questioning the role of sparse coding in the brain. Trends Neurosci 2015; 38: 417–27. 26093844 10.1016/j.tins.2015.05.005

[35] Mairal J , Bach F, Ponce J et al. Online learning for matrix factorization and sparse coding. J Mach Learn Res 2010; 11: 19–60.

[36] Wright J , Ma Y, Mairal J et al. Sparse representation for computer vision and pattern recognition. Proc IEEE 2010; 98: 1031–44.

[37] Pati YC , Rezaiifar R, Krishnaprasad PS. Orthogonal matching pursuit: recursive function approximation with applications to wavelet decomposition. In: Proceedings of 27th Asilomar Conference on Signals, Systems and Computers, 1993, 40–4.

[38] Chen S , Billings SA, Luo W. Orthogonal least squares methods and their application to non-linear system identification. Int J Control 1989; 50: 1873–96.

[39] Beck A , Teboulle M. A fast iterative shrinkage-thresholding algorithm for linear inverse problems. SIAM J Imaging Sci 2009; 2: 183–202.

[40] Rubinstein R , Zibulevsky M, Elad M. Double sparsity: learning sparse dictionaries for sparse signal approximation. IEEE Trans Signal Process 2009; 58: 1553–64.

[41] Yu F , Koltun V. Multi-scale context aggregation by dilated convolutions. arXiv:1511.07122.

[42] Tropp JA. Greed is good: algorithmic results for sparse approximation. IEEE Trans Inf Theory 2004; 50: 2231–42.

[43] Natarajan BK. Sparse approximate solutions to linear systems. SIAM J Comput 1995; 24: 227–34.

[44] Candes E , Romberg J, Tao T. Robust uncertainty principles: exact signal reconstruction from highly incomplete frequency information. IEEE Trans Inf Theory 2006; 52: 489–509.

[45] Donoho DL , Elad M, Temlyakov VN. Stable recovery of sparse overcomplete representations in the presence of noise. IEEE Trans Inf Theory 2005; 52: 6–18.

[46] Gupta S , Agrawal A, Gopalakrishnan K et al. Deep learning with limited numerical precision. In: International Conference on Machine Learning, 2015, 1737–46.

[47] Courbariaux M , Hubara I, Soudry D et al. Binarized neural networks: training deep neural networks with weights and activations constrained to +1 or -1. arXiv:1602.02830.

[48] Netzer Y , Wang T, Coates A et al. Reading digits in natural images with unsupervised feature learning. In: NIPS Workshop on Deep Learning and Unsupervised Feature Learning, 2011.

[49] Krizhevsky A , Hinton G. Learning multiple layers of feature from tiny images. Technical report. University of Toronto, 2019.

[50] Pleiss G , Chen D, Huang G et al. Memory-efficient implementation of densenets. arXiv:1707.06990.

