# Supplementary material for: Towards understanding residual and dilated dense neural networks via convolutional sparse coding
Source: Natl Sci Rev. 2020 Jul 13;8(3):nwaa159. doi: 10.1093/nsr/nwaa159 (PMC8288437; doi:10.1093/nsr/nwaa159)
Supplement: nwaa159_Supplemental_File [file nwaa159_supplemental_file.pdf]

Supplementary Data for "Towards Understanding Residual and Dilated Dense Neural Networks via Convolutional Sparse Coding"

Zhiyang Zhang and Shihua Zhang*
*Email: zsh@amss.ac.cn

I. Convolution and Matrix Multiplication

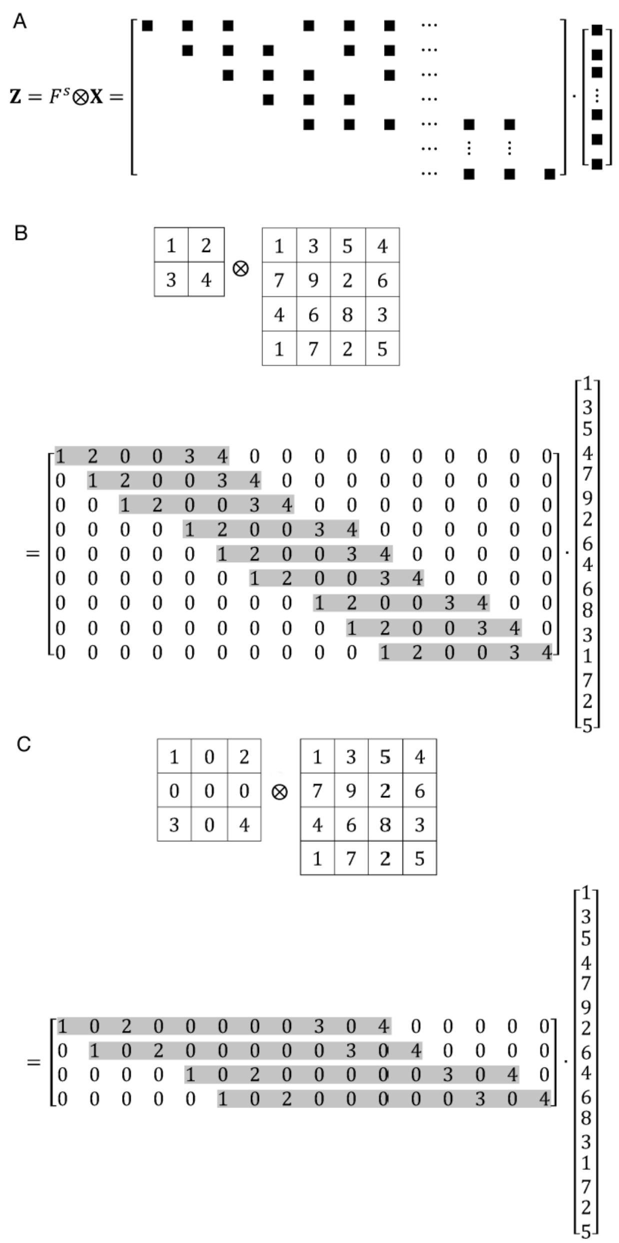

Fig. S1. (A) The matrix-vector multiplication form of the convolution operation. (B) A 2×2 convolution kernel convolves a 4×4 image with dilation scale s=1. (C) A 2×2 convolution kernel convolves a 4×4 image with dilation scale s=2.

II. MSD-CSC and Theoretical Analysis

A. Theorem 1

Theorem 1: In MSD-CSC, all dimensions in Γ_{i-1} will be reconstructed successfully.

Proof: The coding in the i-th layer is $\Gamma_{i-1} = D_i^{s_i}\Gamma_i = \begin{bmatrix} \mathrm{I} & (F_i^{s_i})^T \end{bmatrix} \Gamma_i$, Let $\xi = (\xi_1, \xi_2, \cdots, \xi_n) = D_i \Gamma_i$,

$$f_{MSD-CSC} = \frac{1}{2} \left\| \Gamma_{i-1} - D_i^{s_i} \Gamma_i \right\|_2^2 + \beta \left\| \Gamma_i \right\|_1$$

Suppose the reconstruction in the j-th dimension is considered as unsuccess:

$$\left| \xi_j - \Gamma_{i-1}^{(j)} \right| > 2\beta$$

Let

$$\eta = \left(\Gamma_i^{(1)}, \Gamma_i^{(2)}, \cdots, \Gamma_{i-1}^{(j)} - \xi_j, \cdots, \Gamma_i^{(n)} | \Gamma_i^{(n+1)}, \cdots, \Gamma_i^{(n+m)} \right)^T$$

As illustrated in Fig. SS2. We can obtain:

$$\begin{aligned}
&\frac{1}{2} \left\| \Gamma_{i-1} - D_i^{s_i} \eta \right\|_2^2 + \beta \|\eta\|_1 \\
&= \frac{1}{2} \left\| \Gamma_{i-1} - \begin{bmatrix} I & (F_i^{s_i})^T \end{bmatrix} \eta \right\|_2^2 + \beta \|\eta\|_1 \\
&= f_{MSD-CSC} - \frac{1}{2} \left(\xi_j - \Gamma_{i-1}^{(j)} \right)^2 + \beta \left| \xi_j - \Gamma_{i-1}^{(j)} \right| - \beta |\Gamma_{i-1}^{(j)}| \\
&< f_{MSD-CSC} - \frac{1}{2} \left(\xi_j - \Gamma_{i-1}^{(j)} \right)^2 + \beta \left| \xi_j - \Gamma_{i-1}^{(j)} \right| \\
&< f_{MSD-CSC}
\end{aligned}$$

This indicates that for every solution Γ_i which can not reconstruct all dimensions in Γ_{i-1}, we can always find a solution η. η makes the Lasso problem in MSD-CSC strictly smaller. So, the optimal solution must reconstruct all dimensions in Γ_{i-1}.■

B. Lemma 2

Lemma 2: For a matrix $A \neq 0$, let's assume the matrix AA^T has eigenvalues $\lambda_1, \cdots, \lambda_n$. As a result, the matrix

$$B = \begin{pmatrix} I \\ A \end{pmatrix} \cdot \begin{pmatrix} I & A^T \end{pmatrix} = \begin{pmatrix} I & A^T \\ A & AA^T \end{pmatrix}$$

has eigenvalues $0, \cdots, 0, \lambda_1 + 1, \cdots, \lambda_n + 1$. Here the number of zeros is equivalent to the number of columns of A.

Proof: Assume X is an eigenvector of AA^T corresponding to the eigenvalue λ. Let's consider the vector $X' = \left(\frac{1}{\lambda}A^TX, X\right)^T \neq 0$ and the following equation

$$\begin{pmatrix} I & A^T \\ A & AA^T \end{pmatrix} \cdot \begin{pmatrix} \frac{1}{\lambda}A^TX \\ X \end{pmatrix} = \begin{pmatrix} \frac{1}{\lambda}A^TX + A^TX \\ X + \lambda X \end{pmatrix}$$

$$= (\lambda + 1) \begin{pmatrix} \frac{1}{\lambda}A^TX \\ X \end{pmatrix}$$

Obviously, B has an eigenvalue λ_i+1 and X' is a corresponding eigenvector. So, B has eigenvalues $\lambda_1+1, \cdots, \lambda_n+1$. Let's further consider the trace of B, $\mathrm{tr}(B) = \mathrm{tr}(I) + \mathrm{tr}\left(AA^T\right) = \lambda_1 + \lambda_2 + \cdots + \lambda_n + n$. Then, the sum of the rest eigenvalues is zero because the trace of a matrix is equivalent to the sum of all eigenvalues. In addition, all the eigenvalues of B are nonnegative (A nonzero matrix with the form of MM^T has nonnegative eigenvalues). Taken together, all the rest eigenvalues of B are zeros. ■

$$\begin{bmatrix} \Gamma_{i-1}^{(1)} \\ \Gamma_{i-1}^{(2)} \\ \Gamma_{i-1}^{(3)} \\ \vdots \\ \Gamma_{i-1}^{(j)} \\ \vdots \\ \Gamma_{i-1}^{(n)} \end{bmatrix} \approx \left[\begin{array}{ccccccc|c} 1 & 0 & 0 & \cdots & 0 & 0 & 0 & \\ 0 & 1 & 0 & \cdots & 0 & 0 & 0 & \\ 0 & 0 & 1 & \cdots & 0 & 0 & 0 & \\ \vdots & \vdots & \vdots & \ddots & \vdots & \vdots & \vdots & \left(F_i^{s_i}\right)^T \\ 0 & 0 & 0 & \cdots & 1 & 0 & 0 & \\ 0 & 0 & 0 & \cdots & 0 & 1 & 0 & \\ 0 & 0 & 0 & \cdots & 0 & 0 & 1 & \end{array}\right] \cdot \begin{bmatrix} \Gamma_{i-1}^{(1)} \\ \Gamma_{i-1}^{(2)} \\ \vdots \\ \Gamma_{i-1}^{(j)} - \xi_j \\ \vdots \\ \Gamma_{i-1}^{(n)} \\ \hline \Gamma_{i}^{(n+1)} \\ \Gamma_{i}^{(n+2)} \\ \vdots \\ \Gamma_{i}^{(n+m)} \end{bmatrix}$$

Fig. S2. Illustration of a sparse coding scheme and the recovery role of the identity matrix of the dictionary in the i-th layer of MSD-CSC. Assume the signal in the (i-1)-th layer is n.

III. ALGORITHMS

Algorithm 1 The layered thresholding algorithm

Input:

Signal X

A set of special dictionaries $\{D_i\}$

A set of special thresholding $\{b_i\}$

Thresholding operator $P \in \{S, S^+\}$

Output:

Encoding signals $\left\{\hat{\Gamma}_i\right\}$, $i = 1, 2, \cdots, k$

1. $\hat{\Gamma}_0 \leftarrow X$
2. for $i = 1 : k$ do:
3. $\quad \hat{\Gamma}_i \leftarrow P_{b_i}\left(D_i^T \hat{\Gamma}_{i-1}\right)$

Algorithm 2 MSD-CSC ISTA in the i-th layer

Input:

Signal X, convolution $F_i^{s_i}$, thresholding b_i, parameters c_i width w, unfolding

Output:

Encoding signals $\widehat{\Gamma}_i$

1. $f1 = \mathrm{conv}\left(X, F_i^{s_i}\right)$ //conv denotes convolution
2. $\hat{\Gamma}_i = \mathrm{ReLU}\left(c_i \times \mathrm{concat}(X, f1) + b_i\right)$
3. for $k = 1$: unfolding do:
4. $\quad f1 \leftarrow \hat{\Gamma}_i[1:-w) + (F_i^{s_i})^T \cdot \hat{\Gamma}_i[-w] - X$ //index $-w$ denotes the last w channels
5. $\quad f2 = \mathrm{conv}\left(f1, F_i^{s_i}\right)$
6. $\quad f3 \leftarrow$ concat $(f1, f2)$
7. $\quad \hat{\Gamma}_i \leftarrow \mathrm{ReLU}\left(\hat{\Gamma}_i - c_i \times f3 + b_i\right)$
8. return $\widehat{\Gamma}_i$

Algorithm 3 MSD-CSC FISTA in the i-th layer

Input:

Signal X, convolution $F_i^{s_i}$, thresholding b_i, parameters c_i width w, unfolding, $t_1 = 1$

Output:

Encoding signals $\widehat{\Gamma}_i$

1. $f1 = \mathrm{conv}\left(X, F_i^{s_i}\right)$ //conv denotes convolution
2. $\hat{\Gamma}_i = \mathrm{ReLU}\left(c_i \times \mathrm{concat}(X, f1) + b_i\right)$
3. $\widehat{\Gamma}_i^0 = \widehat{\Gamma}_i^1$
4. for $k = 1$: unfolding do:
5. $\quad t_{k+1} \leftarrow \frac{1+\sqrt{1+4t_k^2}}{2}$
6. $\quad Z \leftarrow \Gamma_i^k + \frac{t_k - 1}{t_{k+1}}\left(\Gamma_i^k - \Gamma_i^{k-1}\right)$
7. $\quad f1 \leftarrow Z[1:-w) + (F_i^{s_i})^T \cdot Z[-w] - X$ //index $-w$ denotes the last w channels
8. $\quad f2 = \mathrm{conv}\left(f1, F_i^{s_i}\right)$
9. $\quad f3 \leftarrow$ concat $(f1, f2)$
10. $\quad \Gamma_i^{k+1} \leftarrow \mathrm{ReLU}\left(Z - c_k \times f3 + b_k\right)$
11. return $\Gamma_i^{1+\text{ unfolding}}$

IV. EXPERIMENTS

All models are trained on a single GPU card: Tesla P40. Generally, Res-CSC needs more training time since the additional term for Res-CSC (Table S1) and MSD-CSC takes more time to train though MSD-CSC has fewer parameters (Table S2). For the MSD-CSC, we note that this situation is not mainly due to the algorithm itself. The key reason is that existing deep learning training software do not support the dilation convolution and dense connection operations well since they assume that all channels of a certain feature map are computed in the same way, and GPU convolution routines such as the cuDNN library assume that feature data is stored in a contiguous memory. Therefore, concatenate operation can be expensive in the current software. Frequent concatenate and split operations are used in MSD-CSC (Fig. S3A). This limits the application of MSD-CSC with more unfolding and layers.

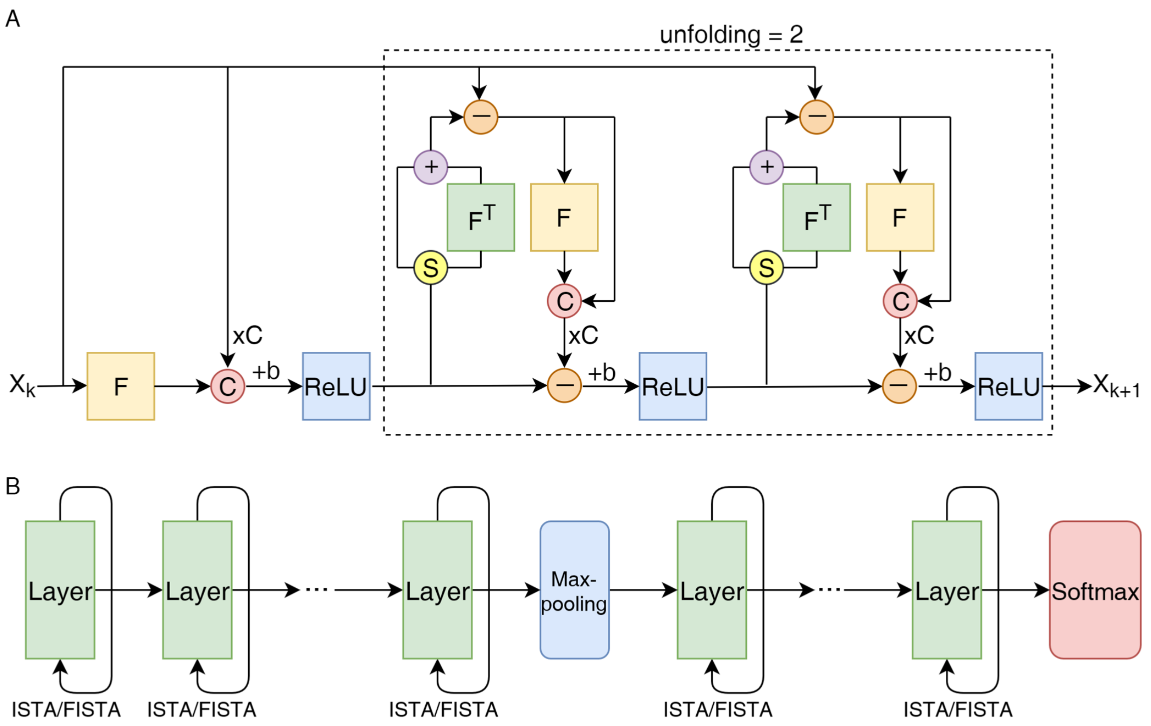

Fig. S3. (A) An illustration shows MSD-CSC ISTA with unfolding = 2. X_k denotes the signal input the k-th layer. F denotes convolution and F^T denotes multiply with the transpose of convolutional matrix, ⓒ denotes concatenate operation, ⓢ denotes split channels, here we split last w channels, w is the number of convolution kernels in this layer. $+$, $-$, $\times$ and ReLU represents their literal meaning. (B) An architecture designed for a classification task. Tensor flow in each layer is illustrated in (A) with ISTA as the forward propagation algorithm.

TABLE S1
COMPUTATIONAL TIME OF RES-CSC AND RESNET ON CIFAR10, CIFAR100 AND SVHN RESPECTIVELY.

Layers	Para	CIFAR10		CIFAR100		SVHN	
		ResNet	Res-CSC	ResNet	Res-CSC	ResNet	Res-CSC
20	0.27M	0.78h	0.89h	0.78h	0.89h	0.67h	1.22h
32	0.46M	0.89h	1.33h	0.89h	1.33h	1.11h	1.89h
44	0.66M	1.06h	2.11h	1.06h	2.11h	1.50h	2.67h
56	0.85M	1.41h	2.78h	1.41h	2.78h	1.89h	3.56h
110	1.70M	2.83h	5.21h	2.83h	5.21h	3.61h	7.11h
218	3.40M	5.21h	9.80h	5.21h	9.80h	7.33h	14.28h

TABLE S2
COMPUTATIONAL TIME OF MSD-CSC AND OTHER CLASSIC CSC MODELS ON CIFAR10, CIFAR100 AND SVHN RESPECTIVELY.

Model	Layer	Para	unfolding	CIFAR10	CIFAR100	SVHN
Feed-Forward	6	4.0M	0	0.59h	0.59h	0.75h
ML-CSC-ISTA	6	4.0M	1	0.67h	0.67h	0.88h
		4.0M	2	0.75h	0.75h	1.08h
ML-CSC-FISTA	6	4.0M	2	0.80h	0.80h	1.12h
MSDNet	6	0.1M	0	0.75h	0.75h	1.17h
	9	0.3M	0	1.08h	1.08h	1.71h
	12	0.6M	0	1.29h	1.29h	1.96h
MSD-CSC-ISTA	6	0.1M	1	2.91h	2.91h	4.46h
			2	5.08h	5.08h	7.75h
	9	0.3M	1	4.25h	4.25h	6.50h
			2	7.41h	7.41h	11.22h
	12	0.6M	1	4.80h	4.80h	7.50h
			2	8.45h	8.45h	12.95h
MSD-CSC-FISTA	6	0.1M	2	5.70h	5.70h	8.70h
	9	0.3M	2	8.25h	8.25h	12.50h
	12	0.6M	2	9.41h	9.41h	14.22h
